# CXCR6 expressing T cells: Functions and role in the control of tumors

**DOI:** 10.3389/fimmu.2022.1022136

**Published:** 2022-10-12

**Authors:** Nesrine Mabrouk, Thi Tran, Ikuan Sam, Ivan Pourmir, Nadège Gruel, Clémence Granier, Joséphine Pineau, Alain Gey, Sebastian Kobold, Elizabeth Fabre, Eric Tartour

**Affiliations:** ^1^ Université ParisCité, INSERM, PARCC, Paris, France; ^2^ Institut Curie, PSL Research University, Department of Translational Research, Paris, France; ^3^ INSERM U830, Equipe labellisée LNCC, Siredo Oncology Centre, Institut Curie, Paris, France; ^4^ Immunology, APHP, Hôpital Europeen Georges Pompidou and Hôpital Necker, Paris, France; ^5^ Division of Clinical Pharmacology, Department of Medicine IV, Klinikum der Universität München, Munich, Germany; ^6^ German Cancer Consortium (DKTK), Partner Site Munich, Munich, Germany; ^7^ Lung Oncology Unit, APHP, Hôpital Européen Georges Pompidou, Paris, France; ^8^ Equipe Labellisée Ligue contre le Cancer, Paris, France

**Keywords:** CXCR6, CXCL16, resident memory T cell, dendritic cell, mucosal vaccination, CAR T cell, immunotherapy

## Abstract

CXCR6 is a receptor for the chemokine CXCL16, which exists as a membrane or soluble form. CXCR6 is a marker for resident memory T (T_RM_) cells that plays a role in immunosurveillance through their interaction with epithelial cells. The interaction of CXCR6 with CXCL16 expressed at the membrane of certain subpopulations of intratumor dendritic cells (DC) called DC3, ideally positions these CXCR6^+^ T cells to receive a proliferation signal from IL-15 also presented by DC3. Mice deficient in *cxcr6* or blocking the interaction of CXCR6 with its ligand, experience a poorer control of tumor proliferation by CD8^+^ T cells, but also by NKT cells especially in the liver. Intranasal vaccination induces CXCL16 production in the lungs and is associated with infiltration by T_RM_ expressing CXCR6, which are then required for the efficacy of anti-tumor vaccination. Therapeutically, the addition of CXCR6 to specific CAR-T cells enhances their intratumoral accumulation and prolongs survival in animal models of pancreatic, ovarian and lung cancer. Finally, CXCR6 is part of immunological signatures that predict response to immunotherapy based on anti-PD-(L)1 in various cancers. In contrast, a protumoral role of CXCR6^+^T cells has also been reported mainly in Non-alcoholic steatohepatitis (NASH) due to a non-antigen specific mechanism. The targeting and amplification of antigen-specific T_RM_ expressing CXCR6 and its potential use as a biomarker of response to immunotherapy opens new perspectives in cancer treatment.

## Introduction

CXCR6 was initially described as a co-receptor for HIV (1) expressed on human memory T cells (2, 3). It is also detected on natural killer (NK) cells (3), NKT cells (4), dendritic cells (DC) (5), alveolar macrophages (6) and innate lymphoid cells (ILC) (7). CXCR6 induction is slow (8 days) after activation (8, 9). IL-15 or antigen exposure followed by TGFβ induce CXCR6 (10) and this effect can be further increased by IL-21 (11).

CXCR6 does not appear to be required in the functionality of CD8^+^T cells (12–14).

Its unique ligand CXCL16 can exist both in a transmembrane and soluble form (2, 15, 16). Membrane CXCL16 acts as an adhesion molecule, whereas its proteolytically cleaved, soluble form acts as a chemoattractant (17). CXCL16 is expressed by epithelial cells, endothelial cells (18) and immune cells such as DC (19, 20).

Several previous studies and reviews highlighted the up-regulation of CXCL16 and/or CXCR6 by tumor cells and their role in tumor growth, migration and invasiveness (21–30). The authors found that CXCL16 is involved in the viability and invasion of tumor cells (22, 31), while the expression of CXCR6 by cancer cells triggers oncogenic pathways associated with cancer progression and metastasis (23, 31). Therefore, in this review, we decided to restrict our focus on the role of CXCR6 expressed by T cells in a tumor context.

## CXCR6 a marker to define T_RM_

Resident memory T cells (T_RM_) are a population of T cells mainly present in tissues and defined usually by the expression of CD103, CD49a and CD69. The CD103 marker interacts with E-cadherin expressed by epithelial cells which explains the role of these cells in immunosurveillance of epithelial tissues (32–34).

CXCR6 is a core marker of T_RM_ in various cancers (6, 35–38). In ovarian and lung cancer, at the protein level, CXCR6 was predominantly expressed on CD8^+^ T_RM_ as compared with intratumoral effector CD8^+^ T cells or circulating T cells (14, 39).

## CXCR6: Role in the differentiation, localization and survival of CD8^+^ T cells in tissues

### Role of CXCR6 in the differentiation of T cells in tissues

**Figure 1:**
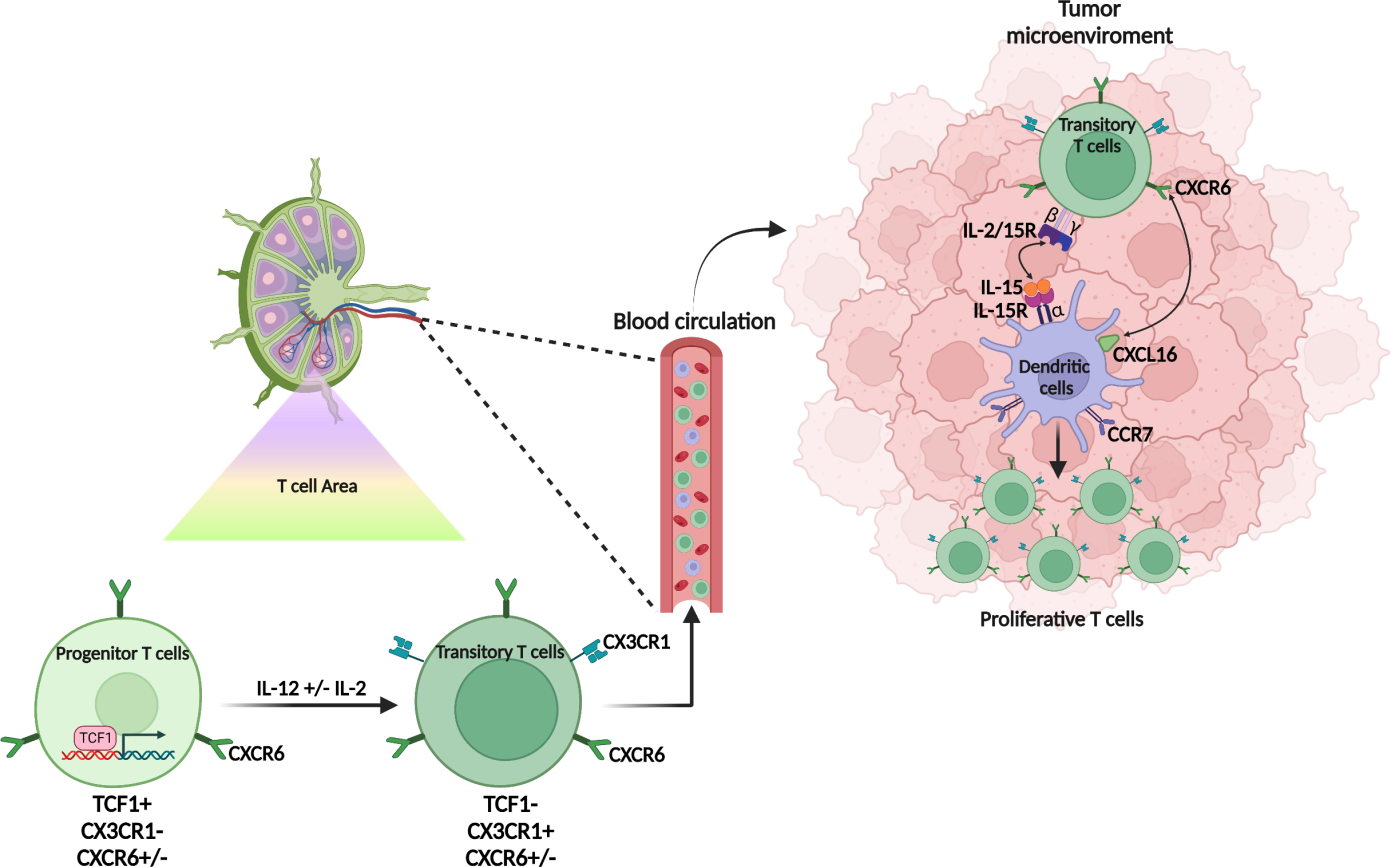
Origin and differentiation of CXCR6^+^ T cells. CXCR6^+^ T cells are scarcely present in the lymph node and CXCR6 weakly expressed by both the progenitor (TCF1^+^) or the transitory (TCF1^-^, CX3CR1^+^) T cell population. In the tumor microenvironment, the transitory T cells express CXCR6 which allows them to interact with the dendritic cell subpopulation (CCR7^+^ DC3) which expresses the membrane form of CXCL16. This contact promotes the interaction of IL-15 also expressed on the membrane of DC3 with the βγ chain of IL-15R expressed on CXCR6^+^ T cells. IL-15 induces the proliferation of CXCR6^+^ T cells and promotes their survival in the tumor microenvironment.

During T cell differentiation program, precursor cells (TCF1^+^) are primed in the lymph node and then migrate into tissues, where they continue their differentiation. CXCR6 is poorly expressed by these TCF1^+^cells. It may explain why in studies using CXCR6^+^ and CXCR6^−^ CD8^+^ T cells, the expression of CXCR6 does not appear to be critical for priming CD8^+^ T cells in lymphoid organs (13, 40). Interleukin-12 promotes loss of TCF-1 and conversion of progenitor into effector CD8^+^ T cells (41). CX3CR1 is induced following the TCF-1^+^ to TCF-1^-^ conversion and characterizes the most highly functional and proliferative CD8^+^ T cell subsets (42, 43). These CX3CR1^+^ TCF1^-^Tbet^+^ CD8^+^ T cells are also called transitory CD8^+^ T cells in mice. They are found in lymph nodes and tissues. CXCR6 up-regulation immediately precedes or accompanies loss of TCF-1 expression in tumor-reactive PD-1^+^ CD8^+^T cells, whose specificity is infered by the expression of PD1, a marker enriched within anti-tumor T cells (**Figure 1**).

*Cxcr6* deficiency in mice neither affects TCF1^+^CD8^+^ T cells in lymph node or tumor microenvironment (TME), nor the CX3CR1 population in lymph nodes. However, it inhibits the expansion of this transient CX3CR1 population in tissues and tumors and the survival of TCF1^-^ populations (40). These elegant works on the role of CXCR6 in T cell differentiation focusing on anti-tumor T cells, do not specify its impact on the differentiation of exhausted T cells and T_RM_. Nevertheless, in the absence of CXCR6, the expression of Tim-3 - a marker of exhaustion - (34) on T cells, is decreased (40) as well as T_RM_ in the TME (12).

### CXCR6 dictates the interaction of T cells with dendritic cells and their subsequent survival

CXCR6 positions CD8^+^ cytotoxic T cells in a distinct perivascular niche of the tumor stroma that is populated by CCR7^+^ DC named DC3 expressing the CXCR6-ligand CXCL16 and trans-presenting the cytokine IL-15 (40). DC3s trans-present IL-15 to CXCR6^+^ TCF-1^-^ effector CD8^+^ T cells to sustain their survival in the TME and avoid AICD (activation-induced-cell-death) (40). *In vitro*, IL-15 expanded CXCR6^hi^ TCF-1^-^ cells (40) (**Figure 1**).

DC3 express CCR7, IL12Rβ, Fascin1 (44, 45) and represent the DC population with the highest expression of CXCL16, CXCL15 and IL-15Rα (40). In humans, this DC3 population has been observed in the TME of breast cancer patients and promotes resident memory CD8^+^ T cell differentiation *via* a TGFβ signaling (see below) (46). CXCR6^+^ CD8^+^ T cells transferred into mice lacking IL-15 or DC do not survive (40).

### Role of CXCR6 in the positioning and survival of CD8^+^ T_RM_ in tissues

In mice, CD8^+^ T cells lacking expression of CXCR6 formed reduced numbers of skin T_RM_ cells, but comparable numbers in the spleen with regards to wild type mice (47).

CXCR6 also positions tumor reactive CD8^+^ T_RM_ with CXCL16^+^ DC clusters in the skin of melanoma-associated vitiligo, which favor their persistence (48).

In an ovarian cancer model, *CXCR6*-deficient mice have less T_RM_ in the tumor (14).

It has also recently been shown that the CXCR6–CXCL16 axis plays a role in the seeding of airway T_RM_ from lung interstitium (49, 50).

## CXCR6 expressing CD4^+^ T cells, NKT and MAIT

In mouse and human, CXCR6 is more expressed in CD8^+^ T cells than in CD4^+^ T cells (40). In the Cancer Genome Atlas (TCGA) database, CXCR6 in tumor tissue correlated highly with CD8 expression and less with expression of CD4 and NK cells (40).

It has been reported that CXCR6^+^ CCR6^+^ CD4^+^ subset is enriched for conventional TH17 molecules (IL-17A, IL-23R, RORγt) and cytotoxic signatures (51, 52), while the CCR6^-^ CXCR6^+^ CD4^+^ T cells expressed IFNγ and GM-CSF, which correspond to a T cell population derived from TH17 cells (52).

CXCR6 plays an essential role in NKT cell development, maturation, homeostatic distribution, glycolipid-induced effector responses, and infiltration into the liver (53, 54). The accumulation of CXCR6^+^ NKT cells in the liver is driven by up-regulated CXCL16 on hepatic sinusoidal endothelial cells, which is induced by gut microbiome-modified bile acids (55). Thus *CXCR6*-deficient mice show profoundly reduced numbers of NKT cells in the liver (53). In addition to regulating NKT cell homing, CXCR6 and CXCL16 have been shown to play a critical role in NKT cell activation in response to glycolipid antigens (15, 53).

MAIT cells are predominantly CXCR6^+^ but do not require CXCR6 for accumulation in lungs after an infection. However, CXCR6 does contribute to long-term retention of MAIT cells in the airway lumen (56).

## Role of CXCR6^+^ T cells in cancer

### Tumor proliferation control

In different preclinical models of melanoma, the absence of CXCR6 led to an acceleration of tumor growth (40, 57). In terms of mechanism, CXCR6 was not essential for extravasation of blood-borne CD8^+^ T cells into tumor tissue (40). Experiments involving the transfer of T lymphocytes expressing or not CXCR6, or blocking the CXCL16-CXCR6 interaction, have shown the requirement of CXCR6-CD8^+^T cells for the anti-tumor function of these T cells (12, 40, 57–59).

In different preclinical liver cancer models, the role of NKT and CD4^+^ T cells producing INFγ and TNFα has also been reported. Thus, in a model of hepatocarcinogenesis*, cxcr6*-deficient mice had significantly more senescent hepatocytes. NKT and CD4^+^ T cells promote the removal of senescent hepatocytes to prevent hepatocarcinogenesis, and this process required CXCR6 (60).

### Role in the control of metastases

Loss of CXCR6 expression on NKT-cells resulted in increased liver metastasis in a murine model (61). *Cxcr6*- deficient mice or CXCL16 neutralizing Ab resulted in an enhanced metastasis to the liver by B16 melanoma cells or Lewis Lung tumor cells (61). In another preclinical model of lung metastases from breast cancer, it has been shown that CXCR6^-^ T effectors are the major subset preferentially egressing the tumor to form distant CXCR6^+^ T_RM_, whereas intratumoral CXCR6^+^ T cells are retained in the tumor (62). Breaking CXCR6-mediated retention in the tumor by anti-CXCL16 treatment resulted in more T cells egressing to the distant lung tissue and a decrease metastatic tumor burden (62).

### Improvement of CAR-T cell therapy

Although CAR-T cell therapy in solid tumors has recently shown its feasibility and clinical signs of effectiveness (63, 64), the efficacy of adoptive cell therapy for solid tumors is hampered by the low infiltration of the tumor after transferred T cells. Recently, it could be shown that T cells expressing a chimeric antigen receptor (CAR) encoding mesothelin and co-transfected with CXCR6 enhanced the efficacy of adoptive cell therapy for pancreatic tumors (65). In mouse models, the addition of CXCR6 to CAR-T cells also enhanced their intratumoral accumulation and sustained their antitumoral activity. Survival was prolonged only when the CAR-T cells co-expressed CXCR6 (65). These results were also reproduced in an ovarian cancer mouse model (65).

Administration of CAR-T cells targeting murine ROR1 – a tumor antigen overexpressed in breast and lung cancer - after lymphodepletion with cyclophosphamide (Cy) transiently controlled lung tumor growth but infiltrated tumors poorly and lost function, as observed in human. Adding oxaliplatin (Ox) to the lymphodepletion regimen activated tumor macrophages to express T cell-recruiting chemokines, resulting in improved CAR-T cell infiltration (66).

At day 2 post-transfer, Ox/Cy enhanced accumulation of CAR-T cells in tumors excised from KP^ROR1^ mice, and this accumulation was partially CXCR6-dependent, as *CXCR6* ^−^/^−^ CAR-T cells showed poorer tumor infiltration compared to their wild type counterparts (66).

### Role in cancer vaccine efficacy

Previous works of our group showed that intranasal vaccination preferentially elicits T_RM_ (67–69). As a continuation of this work, we showed that CXCR6 was preferentially expressed by CD8^+^ T_RM_ after intranasal vaccination in mice with a vector targeting DC (70) and also on intratumoral CD8^+^ T_RM_ derived from human lung cancer (12). We also demonstrated that vaccination of *cxcr6*-deficient mice induces a defect in the lung recruitment of antigen-specific CD8^+^ T cells, mostly in the T_RM_ subsets, responsible for a partial loss of cancer vaccine efficacy (12). Interestingly, intranasal, but not intramuscular vaccination induced higher and more sustained concentrations of CXCL16, compared to other chemokines, in the bronchoalveolar lavage fluid and pulmonary parenchyma (12).

### Role in immunogenicity of radiotherapy

Ionizing radiation appears to increase both in mouse and human the expression of CXCL16 in tumor cells (71) and specifically in breast, colon and prostate cancer (59), favoring the recruitment of effector cells at the tumor site

*Cxcr6*-deficient mice showed reduced infiltration of tumors by activated CD8^+^ T cells and impaired tumor regression following treatment by local irradiation of the tumor (59).

### Pro-tumoral role of CXCR6^+^ T cells

In prostate cancer, inflammatory cytokines derived from the adjacent infiltrating CXCR6-positive T cells stimulate the production of CXCL16 by cancer cells and CXCL16 enhances the growth of CXCR6-expressing cancers and primary T cells (72).

In mice model of Non-alcoholic steatohepatitis (NASH), which causes severe and chronic liver inflammation leading to hepatocarcinoma (HCC), an indispensable role of T cells in liver immunopathology was demonstrated.

CXCR6^+^ CD8^+^ T cells showed killing of cells in an MHC-class-I-independent fashion after signaling through P2X7 purinergic receptors activated by ATP (11).

The exposure of CXCR6^+^ CD8^+^ T cells to ATP led to rapid upregulation of FasL and the death of hepatocytes. The blocking of FasL prevented auto-aggression by CD8 T cells *in vitro* and after adoptive transfer *in vivo*, and ameliorated liver damage in NASH mice (11).

### Role of CXCR6^+^ T cells in clinical response and side effects of anti-PD-1 immunotherapy

In a preclinical model of melanoma and colorectal cancer, anti-PD-1 treatment significantly increased CXCR6 expression on infiltrating CD8^+^ T cells (57). Interestingly, the percentages of intratumoral CD8^+^ T cells and cytokines production, as well as the efficacy of therapy, were rapidly decreased in *cxcr6^−^/^−^* mice treated with PD-1 blockade therapy (57).

In contrast, when given prophylactically in a mice model of NASH, anti-PD1 treatment led to an increase in the incidence of NASH induced HCC and in the number and size of tumor nodules, which correlated with increased hepatic PD1^+^ CXCR6^+^ CD8^+^ T cells and TNF^+^ T cells (73). The increase in HCC triggered by anti-PD1 treatment was prevented by depletion of CD8^+^ T cells or TNF neutralization (73). These results may seem contradictory to the clinical data on the efficacy of anti-PD1 in liver cancer. In fact, a meta-analysis of 1,600 patients revealed that immunotherapy based on PD-1-PD-L1 blockade did not improve survival in patients with non-viral HCC (73).

CXCR6^+^ T cells have also been implicated in the side effects of immunotherapy. Thus, a striking accumulation of CXCR6^+^ CD8^+^ T cells with highly cytotoxic and proliferative states is observed in checkpoint inhibitor-induced colitis (74). Interestingly, administration of an anti-CXCL16 mAb reduced inflammation in a chemically induced experimental colitis model (75).

### CXCR6, a prognostic and predictive biomarker for cancer immunotherapy

In most cancers (melanoma, head and neck cancer, lung adenocarcinoma, and breast cancer), patients with high CXCR6 expression had a greater survival probability (40). An exception concerns liver cancer, where CXCR6 and its cognate ligand CXCL16 have been associated with higher HCC invasiveness, poor prognosis and predictor of recurrence (18, 28).

The good prognosis related to CXCR6 expression is associated with CD8^+^ T cell infiltration often corresponding to a T_RM_ phenotype.

Similarly, high expression of CXCR6 in colorectal cancer was associated with a good prognosis and positively correlated with the expression of CD8 in tumor (57).

Analysis of The Cancer Genome Atlas (TCGA) for ovarian cancer revealed CXCR6 expression to be associated with CD103 and increased patient survival (14).

Interestingly, CXCR6 constitutes 1 of 18 genes that are developed and validated as a clinical grade biomarker to predict the response to anti-PD-1 therapy in various cancers (76, 77).

## How to elicit CXCR6^+^ T cells

**Figure 2:**
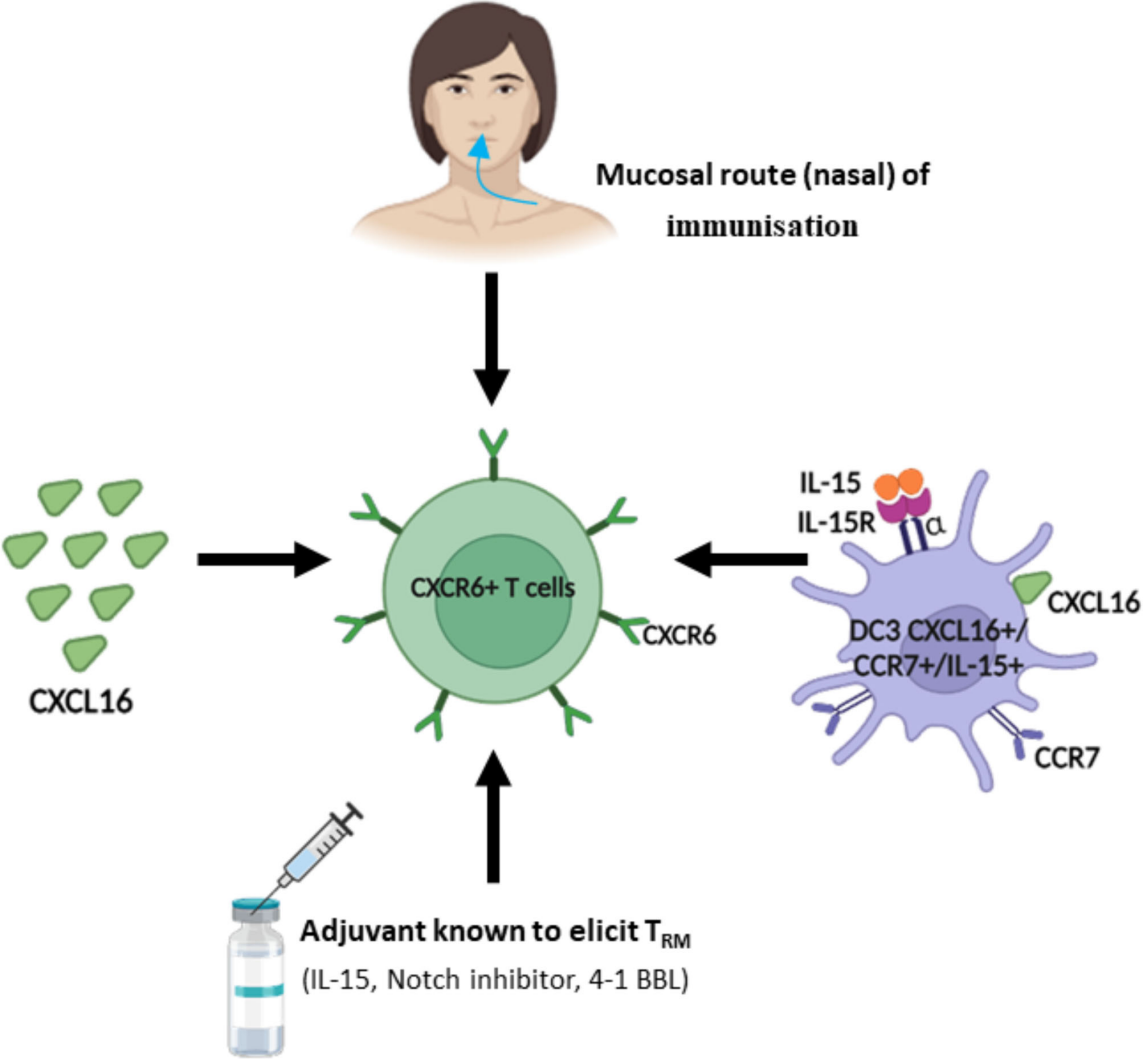
Strategy to regulate and induce CXCR6^+^ T cells.In light of the role of CXCR6^+^ T cells in tumor control, different strategies have been proposed to induce or increase their recruitment in the tumor microenvironment. As the majority of CXCR6^+^ T cells are included in the resident memory T cell population (T_RM_), many strategies are common with those aimed at inducing T_RM_.

To induce CXCR6^+^ T cells in the lungs and head and neck tissue, we have shown that the nasal route of immunization appears to be the most effective in different experiments (12). These T cells had a phenotype of CD8^+^ T_RM_. Similar results on the value of this mucosal route of immunization have been reported by different groups (78–80) (**Figure 2**).

This mucosal CD8^+^ T cell response can be maintained for several months. Interestingly, not all mucosal routes are equivalent in inducing these CXCR6^+^ T cells. For example, the oral route of immunization induces mainly the chemokine CCR9 but not CXCR6 (79). We and other groups have shown that this nasal immunization, but not the systemic routes (s.c or IM) induced CXCL16 in the lungs likely produced by epithelial cells and DC and promoting their interaction with CXCR6^+^ T cells (12, 78, 80). This induction of endogenous production of CXCL16 may explain conflicting results of CXCL16 administration as a vaccine adjuvant (12, 78, 79).

The subcutaneous route results in a small increase of CXCR6 in lymph node T cells (81). Parenteral routes are poorly effective in inducing these CXCR6^+^ T cells in the lungs (78).

*In vitro*, DC3 are able to induce CD8^+^ T_RM_ expressing CXCR6 and promote their expansion (40). Some adjuvants (IL-15, 4-1BBL, Notch inhibitors, …) known to increase CD8^+^ T_RM_ could be evaluated for their ability to induce CXCR6 (69) (**Figure 2**).

## Discussion and conclusion

CXCR6 can be considered as a new class of chemokine receptor, whose main role could be to allow the positioning of T cells in close interaction with cells expressing CXCL16 at the membrane such as DC3, and so to promote T cell differentiation. The adhesion role of CXCL16 known for many years, has been strengthened recently and the CXCL16-CXCR6 interaction seems to participate in a synapse including IL-15 and IL-15Rα to promote tissue T cell differentiation, particularly toward a T_RM_ phenotype (40, 48).

An ambivalent role of CXCR6^+^ T cells in tumor control has been reported. Thus, in many models, CXCR6^+^ CD8^+^ T cells of resident phenotype participate in the control of primary tumor proliferation and metastasis (12, 57, 62). On the contrary, in NASH models with significant chronic inflammation, CXCR6^+^ P2X7^+^ T cells are able to destroy hepatocytes in a non-MHC-restricted manner which distinguishes them from classical CD8^+^ T_RM_ (11, 73).

This ambivalent role of CXCR6^+^ T cells in the regulation of tumor growth has also been established for other subpopulations of T cells such as TH17 (82–84) and for myeloid cell subpopulations (85–87). It may be explained by differences in fine phenotype and function for the same immune sub-population and may also depend on the tumor stage and the acute or chronic inflammatory context (88).

The mucosal nasal route of immunization preferentially induces these CXCR6^+^ T cells in the nasal and pulmonary mucosa as already reported for T_RM_ which also express CXCR6 (12, 78, 79). The induction of CXCL16 by this immunization route may explain this specific recruitment of CXCR6^+^ T cells by this mucosal pathway.

In most models, these CXCR6^+^ T cells belong to the subpopulation of T_RM_. However, NKT cells also express CXCR6, which seems to play a major role in the development of these cells, especially in the liver. CXCR6^+^ NKT cells regulate hepatocarcinogenesis and metastasis formation and control hepatitis (53–55).

Finally, from a clinical perspective, CXCR6 expression in CAR^+^ T cells has improved their efficacy in various preclinical solid tumors and might indicate a track to translation of the approach (65, 66). Anti-PD-1 antibodies which increase T_RM_ also modulates CXCR6 (57) and in fact, molecular signatures including CXCR6 appear to predict response to immunotherapy (76, 77), underpinning the high relevance of the pathway.

## Author contributions

NM and ET designed the literature search and wrote the article with input from all authors. NM, ET, and NG designed the figures. All authors contributed to the article and approved the submitted version.

## Funding

This research was funded by grants from Fondation ARC pour la recherche sur le cancer (Grant number SIGN’IT20181007747 and PGA12019110000946_1581), INCA (contract 2019-1-PLBIO-05-1)(PLBIO22-147), Foncer contre le Cancer, SIRIC CARPEM, Labex Immuno-Oncology, Ligue contre le cancer (MucoRNAVax), IDEX Université de Paris (Grant number AMI-CD27CD70BLOC), Inserm Transfert (Grant number: MAT-PI-20278-1). This study was supported by the Marie Sklodowska-Curie Program Training Network for Optimizing Adoptive T Cell Therapy of Cancer funded by the H2020 Program of the European Union (Grant 955575, to SK); by the Hector Foundation (to SK); by the International Doctoral Program i-Target: Immunotargeting of Cancer funded by the Elite Network of Bavaria (to SK); by Melanoma Research Alliance Grants 409510 (to SK); by the Else Kröner-Fresenius-Stiftung (2021_EKFK_01, to SK); by the German Cancer Aid (to SK); by the Ernst-Jung-Stiftung (to SK); by the LMU Munich’s Institutional Strategy LMUexcellent within the framework of the German Excellence Initiative (to SE and SK); by the m4-award of the Bavarian Ministry for economical affairs (to SK), by the Go-Bio-initiative (to SK), by the Bundesministerium für Bildung und Forschung (to SK); by the European Research Council Grant 756017, ARMOR-T (to SK); by the Wilhelm-Sander-Stiftung (to SK), by the German Research Foundation (DFG) (to SK); by the SFB-TRR 338/1 2021–452881907 (to SK); by the Fritz-Bender Foundation (to SK) and by the Deutsche José-Carreras Leukämie Stiftung (to SK).

## Acknowledgments

The figures were created with BioRender.com.

## Conflict of interest

The authors declare that the research was conducted in the absence of any commercial or financial relationships that could be construed as a potential conflict of interest.

## Publisher’s note

All claims expressed in this article are solely those of the authors and do not necessarily represent those of their affiliated organizations, or those of the publisher, the editors and the reviewers. Any product that may be evaluated in this article, or claim that may be made by its manufacturer, is not guaranteed or endorsed by the publisher.
